# Methyl Donor Micronutrients that Modify DNA Methylation and Cancer Outcome

**DOI:** 10.3390/nu11030608

**Published:** 2019-03-13

**Authors:** Abeer M. Mahmoud, Mohamed M. Ali

**Affiliations:** 1 Department of Physical Therapy, College of Applied Health Sciences, University of Illinois at Chicago, 1919 W. Taylor St. (AHSB), Room 433, Chicago, IL 60612, USA; mali37@uic.edu; 2 Integrative Physiology Laboratory, College of Applied Health Sciences, University of Illinois at Chicago, Chicago, IL 60612, USA

**Keywords:** methyl donors, nutrients, DNA methylation, cancer, folate, Vitamin B, choline, betaine, methyltransferase, dietary

## Abstract

DNA methylation is an epigenetic mechanism that is essential for regulating gene transcription. However, aberrant DNA methylation, which is a nearly universal finding in cancer, can result in disturbed gene expression. DNA methylation is modified by environmental factors such as diet that may modify cancer risk and tumor behavior. Abnormal DNA methylation has been observed in several cancers such as colon, stomach, cervical, prostate, and breast cancers. These alterations in DNA methylation may play a critical role in cancer development and progression. Dietary nutrient intake and bioactive food components are essential environmental factors that may influence DNA methylation either by directly inhibiting enzymes that catalyze DNA methylation or by changing the availability of substrates required for those enzymatic reactions such as the availability and utilization of methyl groups. In this review, we focused on nutrients that act as methyl donors or methylation co-factors and presented intriguing evidence for the role of these bioactive food components in altering DNA methylation patterns in cancer. Such a role is likely to have a mechanistic impact on the process of carcinogenesis and offer possible therapeutic potentials.

## 1. Introduction

Cancer is an outcome of aberrant genetic and epigenetic events. Epigenetic mechanisms are responsible for regulating gene expression without changing the DNA sequence. These mechanisms mainly include chromatin remodeling, histone modification, and DNA methylation, the latter being the most investigated mechanism and the focus of the current review [1]. The process of DNA methylation includes the addition of methyl groups to the cytosine residues; a biological process that depends on the availability of methyl groups and accordingly the function of methyl donors and acceptors [2]. Micronutrients such as folate, choline, betaine, vitamin B12, and other B vitamins contribute to DNA methylation as methyl donors and co-factors [3]. Therefore, the status of these nutrients might correlate with DNA methylation and offer potential preventive and therapeutic targets in pathological conditions such as cancer where aberrant DNA methylation is frequently observed.

It is widely accepted that DNA methylation profiles are dynamic and prone to modifications in response to normal development and aging, environmental factors, and pathological conditions [4,5]. For example, DNA undergoes progressive global hypomethylation and gene-specific hypermethylation as individuals age, leading to genomic instability or gene-specific suppression [4]. A similar pattern has been observed in cancer where DNA is globally hypomethylated while tumor suppressor genes are hypermethylated compared to normal tissues [6]. Whether these epigenetic patterns are a cause or an outcome of cancer is not entirely understood. Yet, cancer could be prevented through changes in diet or lifestyle that might be attributed to the dynamic and adaptable nature of cancer-associated epigenetic processes, particularly, DNA methylation.

In the present review, we summarized preclinical and clinical studies that provide intriguing evidence on the interrelation between nutritional methyl donors and carbon one metabolism co-factors and cancer through regulating DNA methylation patterns. Additionally, this review characterizes the differential effects of micronutrient methyl donors on global versus gene-specific DNA methylation and the effect of variables such as health status, source of biological samples that were analyzed, mode (dietary versus supplemental) and quantity of nutrient exposure, and other confounders in an effort to unravel potential sources of discrepancies in the literature

## 2. DNA Methylation

DNA methylation is an epigenetic mechanism that is essential for regulating gene transcription. This mechanism is mediated via the addition of a methyl group to the cytosine residues in a cytosine-guanine (CG) pair generating 5-methylcytosine. In the intergenic regions and repetitive sequences of the human genome, CpG sites are sparse and mostly methylated. Hypomethylation of CpG sites in these regions may lead to genomic instability and loss of gene imprinting, which eventually result in the development of neoplastic cells [7]. On the other hand, gene promoters are rich in CpG sites that are sometimes densely packed forming what is known as CpG islands. These islands are mostly unmethylated in order to allow gene transcription. Aberrant hypermethylation of these CpG sites may silence the expression of genes that are critical to cell homeostasis, DNA integrity, or genome stability, resulting in cancer development and progression [8].

The process of DNA methylation is catalyzed by a group of enzymes called DNA methyltransferases (DNMTs), namely, DNMT1, DNMT2, DNMT3a, DNMT3b, and DNMTL. DNMT1 maintains DNA methylation during cell replication while the rest of the DNMT family, mainly DNMT3a and DNMT3b, are responsible for de novo DNA methylation [9].

DNMTs that are responsible for *de novo* DNA methylation are highly expresses in developing embryos than adult tissues; yet, there is growing evidence that they play a role in maintaining DNA methylation patterns. For instance, combined genetic deletion or silencing of DNAMT1 and DNMT3b reduced DNA methylation to a greater extent than deleting or silencing either genes alone, supporting the critical role of de novo DNMTs in maintaining DNA methylation [10,11,12]. On the other hand, some studies suggested that DNMT1 is required for de novo DNA methylation. For example, a study by Liang et al. [13] showed that DNMT3a and DNMT3b did not induce de novo DNA methylation efficiently in mouse embryonic stem cells in the absence of DNMT1 gene. Other studies have supported this co-operativity between DNMTs in de novo DNA methylation [14,15,16]. The process of methyl transfer starts by non-specific binding of DNMTs to DNA followed by recognition of specific DNA sites and recruitment of the methyl group donor, S-adnosylmethionine. DNMTs incorporate the donated methyl group into carbon 5 of the cytosine residue followed by a release of the DNMT enzyme and s-adenosylhomocysteine [16].

When it takes place at gene promoters, DNA methylation results in transcriptional repression either via interfering with the binding of transcription activating factors or by recruiting transcriptional repressors such as methyl-binding proteins, histone deacetylases (HDACs), and histone methyltransferases that reduces chromatin accessibility [17]. Absent or inactive DNMTs, mainly DNMT1, will induce passive demethylation of the CpG sites and subsequently aberrant gene expression [18]. DNA hypomethylation can also be induced via active demethylation by the ten-eleven translocation methylcytosine dioxygenase family of enzymes (TETs) that helps create a balanced methylation profile in the human genome [19]. TET enzymes oxidize 5-methylcytosine (5-mC) to 5-hydroxy-mC (5-hmC), which is modified through several suggested mechanisms including deamination and decarboxylation that ultimately lead to base excision repair and replacement with an unmethylated cytosine [20]. TET1 is the most prominent member of the TET family, and previous studies showed that knockdown of TET1 results in increased global methylation in mice [21]. Other suggested mechanisms for active DNA demethylation include: (1) base excision repair via DNA glycosylase either by directly acting on 5-mC residue or following 5-mC deamination and conversion into Thymine; (2) oxidative or hydrolytic removal of the methyl group; or (3) nucleotide excision repair system that severs methylated CpG dinucleotides. Current efforts are underway to study the role of DNA active demethylation in cancer and developmental diseases [22].

## 3. DNA Methylation in Cancer

The two main salient features of aberrant DNA methylation in cancer are 1) global DNA hypomethylation that takes place in the intergenic regions and repetitive DNA sequences and 2) local DNA hypermethylation in CpG islands located in specific gene promoters [23]. The latter phenomenon is induced by de novo DNA methylation that is mediated via DNMT3a and DNMT3b and is accompanied by a suppressed transcription of corresponding genes [24]. Several studies have shown that DNA hypermethylation in cancer cells targets tumor suppressor genes explicitly, resulting in growth selection and uncontrolled cell proliferation [25,26,27,28]. Many tumor suppressor genes are inactivated by this mechanism in cancer such as the adenomatous polyposis coli (APC) [29], retinoblastoma (Rb) [30], Von Hippel-Lindau (VHL), BRCA1 [31], and several other genes that are involved in DNA repair (MGMT; O-6-Methylguanine-DNA Methyltransferase), cell cycle progression (p16^INK4a^, p15^INK4b^), apoptosis (DAPK; death-associated protein kinase-1), or antioxidation (GSTP1; Glutathione S-Transferase P-1) [32]. The extent to which DNA de novo methylation contributes to cancer development and progression varies among different types of cancer; very high in colon cancer, while rare in brain tumors [33].

There is also growing evidence indicating that global hypomethylation may lead to genome instability and DNA breakage [4]. Furthermore, global hypomethylation may be accompanied by loss of imprinting of some oncogenes leading to cancer development such as insulin-like growth factor 2 (IGF-1) in colon cancer [34]. The oncogenes that are linked to cancer and activated via hypomethylation are protease urokinase, mesothelin, cancer-testis genes, claudin4, S100A4, heparinase, and the proopiomelanocortin gene [35]. Global DNA hypomethylation in cancer cells has been suggested to be attributed to one of the following causes: (1) a discoordination between DNA replication in cancer cells and DNMT-1 activity; (2) a natural selection of hypomethylated DNA patterns accompanying overexpression of specific oncogenes or genomic instabilities that facilitate cancer cell growth and expansion; or (3) a consequence of chromatin dysregulation and nuclear disorganization that occur during cancer progression [36].

Under normal conditions, cells possess standard methylation profiles by maintaining a balance between DNA methylation and demethylation processes. This balance is disturbed under pathological conditions such as inflammation, oxidative stress and cancer resulting in diverse phenotypes [37]. Due to the dynamic and reversible nature of the DNA methylation process, it has been viewed as an attractive target for cancer therapy. The de novo DNA methylation in cancer has been successfully targeted via the DNMT potent inhibitor, 5-aza-2′-deoxycytidine (5-AZA) in experimental studies and in the clinical settings for treating leukemia [38]. Treatment with 5-AZA reduced DNMT3b in breast cancer cells and restored the expression of TSGs such as RASSF1A (Ras association domain family member 1) in hepatocellular carcinoma, P53 in melanoma, and cyclin-dependent kinase-2B (CDKN2B) in myelodysplastic syndrome [39,40,41,42].

There is a cumulative body of evidence indicating DNA methylation adaptability to environmental factors including diet and nutritional elements [43,44]. Nutrients have been shown to modify DNA methylation either globally or at specific CpG sites by inducing the formation of methyl donors, acting as co-enzymes, or modifying DNMT enzymatic activity [45]. The interaction between nutrients and epigenetics has been referred to as “Nutri-epigenomics”, which is an emerging and promising field that offers opportunities for future applications of bioactive nutrients as epigenetic modifiers in diseases where aberrant DNA methylation occurs [46,47].

## 4. Effects of Nutrients and Bioactive Food Components on DNA Methylation

**Figure 1:**
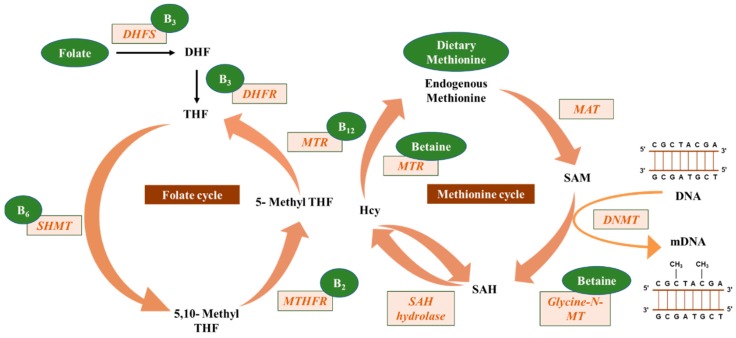
Micronutrient methyl donors that are involved in the one carbon metabolism and subsequently in DNA methylation. Dietary folate is converted to dihydrofolate (DHF) via the dihydrofolate synthase (DHFS) enzyme then to tetrahydrofolate (THF) by the dihydrofolate reductase (DHFR) enzyme; in both steps, vitamin B_3_ (B_3_) acts as a co-factor. THF is then converted to 5,10-methyl THF via the enzyme serine hydroxymethyltransferase (SHMT) that has vitamin B_6_ (B_6_) as a coenzyme. This reaction is followed by a reduction of 5,10-methyl THF to 5-methyl THF via the enzyme methylenetetrahydrofolate reductase (MTHFR) and the co-enzyme, vitamin B_2_ (B_2_). At the end of this cycle, 5-methyl THF is transformed back to THF by the enzyme 5-methyltetrahydrofolate-homocysteine methyltransferase (MTR) that utilizes vitamin B_2_ as a co-enzyme. The same enzyme, MTR, converts homocysteine (Hcy) to methionine. Betaine acts as an indirect methyl donor for the latter reaction. Methionine, whether it is endogenously synthesized or diet-derived is critical for the synthesis of S-adenosylmethionine (SAM), which acts as a DNA methyltransferase (DNMT) cofactor and a universal methyl-donor for DNA methylation. The enzyme that catalyzes this reaction is methionine adenosyltransferase (MAT). Glycine N-methyltransferase (Glycine N-MT) converts SAM to s-adenosylhomocysteine (SAH), which could be reversibly converted to Hcy via the enzyme SAH hydrolase. Finally, the activated DNMT enzyme will catalyze the transfer of a methyl group to carbon 5 of cytosines in the DNA to produce methylated DNA (mDNA).

One of the proposed mechanisms by which nutrients may modify DNA methylation is by participating in a cellular process known as “one-carbon metabolism” that provides methyl groups for biological methylation of DNA, protein, or phospholipids [48]. One carbon metabolism is a process that is involved in amino acid and nucleotide metabolism and comprises a group of biochemical reactions that are catalyzed by a unique set of enzymes and coenzymes (Figure 1). This set of reactions is referred to as one-carbon metabolism since they transfer one-carbon groups from donors to protein or DNA. During this process, cyclical chemical reactions take place in mammalian cells where nutrients such as folate, vitamin B12, vitamin B6, betaine, choline, and methionine play a significant role as co-factors or methyl acceptors or donors [49]. This process starts with a one-carbon transfer from the amino acid serine to tetrahydrofolate; the latter is a form of vitamin B-9 that is naturally present in many food sources and is essential for the nucleic acid synthesis and other vital biological processes in the body. This reaction is catalyzed by the enzyme serine hydroxymethyltransferase, a vitamin B6-containing enzyme. The outcome of this reaction is 5,10 methylene tetrahydrofolate, which, in turn, is transformed into 5-methyl tetrahydrofolate via the enzyme tetrahydrofolate reductase, a vitamin B2-containing enzyme. The product 5-methyl tetrahydrofolate is the primary methyl donor for the reaction that remethylates, homocysteine into methionine. The latter reaction is catalyzed by the enzyme methionine synthase, which contains vitamin B12 as a co-factor. Methionine is then converted to s-adenosylmethionine, the DNMT cofactor and the universal methyl donor for all the biological methylation process inside mammalian cells including DNA methylation [2,50,51].

The negative feedback mechanism in this cycle is catalyzed by two enzymes, glycine N-methyltransferase that converts s-adenosylmethionine to s-adenosylhomocysteine and s-adenosylhomocysteine hydrolase that converts s-adenosylhomocysteine to homocysteine [52]. This reaction is considered as a negative regulator of the DNA methylation process since s-adenosylhomocysteine binds to DNMTs with a higher affinity than s-adenosylmethionine resulting in an inhibition of the DNMT activity. The conversion of s-adenosylhomocysteine to homocysteine is a reversible chemical reaction, and accordingly high levels of homocysteine in the body are associated with reduced DNMT activity. This negative regulation of the “one-carbon metabolism” cycle is resolved by the re-methylation of homocysteine to methionine via the folate-dependent pathway or the diet-derived betaine or choline in the liver and kidney [53]. The alternative pathway for homocysteine metabolism is through transsulfuration to cystathionine, via cystathionine β-synthase. The latter is further metabolized to cysteine via the enzyme, cystathionase. Both reactions utilize the cofactor pyridoxal-5′-phosphate [54].

**Figure 2:**
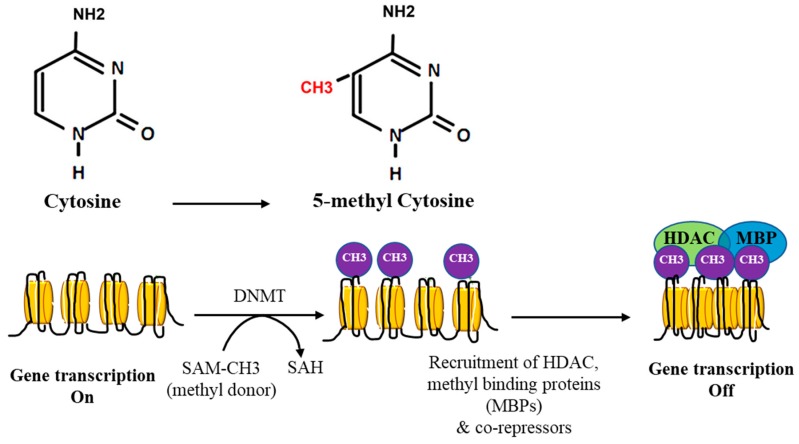
Schematic representation of DNA methylation and its effect on gene transcription. DNA methyltransferase (DNMT) converts cytosine to 5′methyl-cytosine. The process involves the transfer of a methyl group from S-adenosylmethionine (SAM) to the cytosine resulting in the conversion of DNA to methylated DNA and methylated SAM to non-methylated S-adenosylhomocysteine (SAH). DNA methylation recruits histone deacetylase (HDAC), methyl binding proteins (MBPs), and other transcription repressing factors. These modifications result in closed chromatin conformation, inaccessibility to transcriptional machinery, and eventually gene silencing.

The “one-carbon metabolism” cycle is an example of how levels of nutrient exposure impact DNA methylation in the human body (Figure 2). In this example, nutrients either maintain or change the balance between s-adenosylmethionine and s-adenosylhomocysteine, which, in turn, regulate the availability of methyl donors and the activity of DNMTs [2]. There is a growing body of evidence indicating that nutrients, such as the green tea polyphenol, epigallocatechin-3-gallate, or the soy isoflavone, genistein, are capable of inhibiting DNMT activity directly and competitively [49]. Furthermore, dietary supplements that have alpha-ketoglutarate may affect the activity of the active demethylation enzymes, TETs [55]. In the current review, we will discuss reported studies and observations for the mechanism and role of dietary methyl donors in modifying DNA methylation and subsequently cancer risk or progression. These studies are summarized in Table 1 and Table 2.

## 5. Micronutrients and DNA Methylation and Their Impact on Cancer

### 5.1. Folate

Folate naturally occurs in a wide variety of foods. Rich sources include leafy green vegetables, beans, peas, lentils, fruits like lemons, bananas, and melons, and fortified and enriched products, such as breads and cereals [112]. Folate is essential for several biological cell processes such as DNA synthesis and methylation, production and maintenance of new cells, and amino acid metabolism [50]. In several studies, folate supplementation has been shown to increase DNA methylation. For example, in a clinical trial conducted by Wallace et al. [56], folate supplementation increased DNA methylation of two proto-oncogenes in colorectal mucosa, estrogen receptor alpha (ER-α) and secreted frizzled-related protein-1 (SFRP-1). Similarly, in an in vitro model of neuroblastoma cells, Li et al. [113] have shown that treatments with folic acid (20 and 40 mmol/L) increased s-adenosylmethionine to s-adenosylhomocysteine ratio and subsequently downregulated the abnormally phosphorylated tau protein by inhibiting the demethylation of its regulator, PP2A (protein phosphatase 2A). Additionally, animal studies suggest that folate deficiency or supplementation modifies the methylation status of DNA promoter in genes that are critical in carcinogenesis. Folate-rich diet increased DNA methylation of genes such as p16 [114] in mouse colon and protooncogenes such as PDGF-B (platelet-derived growth factor-B), Ras (rat sarcoma), and survivin in a mouse model of gliomagenesis [115].

Conflicting results regarding the effect of folate status on DNA methylation have been reported. For example, global DNA hypomethylation was reported in three different murine studies in response to either a folate-deficient or a folate-rich diet provided during gestation, lactation, or after weaning [116,117,118]. Another study showed that a folate-deficient diet reduced tumor size in a colorectal cancer mouse model with no effect on global or gene-specific DNA methylation. However, this reduction in tumor size was only observed when the folate-deficient diet was administered after tumor development, and no effect was observed when folate deficiency was induced before tumor development [119]. In a study by Kotsopoulos et al. [120], folate-deficient diet increased DNA methylation in rat liver when administered post weaning and this effect continued through adulthood; no changes in DNA methylation were observed when either a folate-deficient or a folate-rich diet was administered at puberty. These findings may suggest that the dietary level of folate is not the only determinant for DNA methylation status and that other confounding factors might modulate the role of folate as a methyl donor.

Similarly, data from human studies that assessed the contribution of folate deficiency or folate supplementation on cancer risk or progression is inconsistent and varies significantly based on factors such as folate dose, mode of intake (dietary versus supplementary), stage of development during exposure (prenatal versus postnatal), and the pathological status (normal versus neoplastic) of tissues [121,122,123,124]. Other factors such as age, genetic and epigenetic background, alcohol intake, and comorbidities might influence the outcome in folate consumers [125,126].

Global DNA hypomethylation and gene-specific hypermethylation are significant characteristics of some cancers such as colorectal and prostate cancer and reversing these epigenetic changes has been of great interest for cancer researchers [127,128]. Folate intake has been reported to correct global DNA hypomethylation and imprint proto-oncogenes such as H-Ras in patients with colorectal cancer [129]. In fact, several studies that investigated the effect of folate on cancer focused on colorectal cancer. For example, an observational study by Giovannucci et al. [83] reported an inverse association between high folate intake and the risk of colorectal adenoma in men and women from the Health Professional Follow-up Study and the Nurses’ Health Study, respectively. Furthermore, findings from the NHANES (National Health and Nutrition Examination Survey) Epidemiologic Follow-up Study (NHEFS) support the association between folate intake and reduced risk of colon cancer [84]. In this study, men who consumed more than 249 μg/day had a lower risk of colon cancer (Relative Risk (RR): 0.40). Similarly, a prospective study of 88,758 women reported reductions in colon cancer risk in women who consumed more than 400 μg/day (RR: 0.81), especially those with a family history of colon cancer (RR: 0.48) [85]. These findings were reproduced by Stevens et al. in 43,512 men and 56,011 women from the Cancer Prevention Study II Nutrition Cohort. Folate intake was also shown to reduce prostate cancer risk in the American Cancer Society Cancer Prevention Study II Nutrition Cohort [86]. 

However, conflicting evidence has emerged, indicating that the mechanisms associated with folate effect on DNA methylation are more complex than previously thought and confounded by other dietary, genetic, or tissue-related factors. For example, a prospective nested case-control study of 331 cases and 662 matched controls in the population-based Northern Sweden Health and Disease Study demonstrated an association between low plasma levels of folate and reduced risk of colon cancer [87]. This study concluded that low circulating levels of folate are protective against colon cancer. Findings from other epidemiological studies were inconsistent; only five out of seven prospective studies [84,88,89,90,91,92,130] and seven out of 11 case-control studies [93,94,95,96,97,98,99,100,101,131,132] have reported a protective effect of folate intake against colon cancer. This discrepancy motivated researchers to conduct meta-analyses of published observational studies to provide an overall estimate of the association between folate intake and colorectal cancer risk. A meta-analysis by Sanjoaquin et al. [133] indicated that dietary folate has a more protective effect on colorectal cancer than supplemental folate. However, confounding factors such as gender, other dietary factors, and alcohol consumption modify the association. A relatively recent meta-analysis reported a lack of association between folate supplementation and total cancer incidence including colorectal cancer, lung cancer, prostate cancer, or breast cancer [134]. Observational studies that assessed the association between folate status and global DNA hypomethylation in cancer patients have also yielded mixed findings. An association between global DNA hypomethylation and increased risk of colon, cervical, and bladder cancer was established in these studies [57,58,59]. However, a role of low folate status (intake or blood levels) in this association could not be consistently found. A significant association between folate status and DNA hypomethylation was reported by Piyathilake et al. [60] in their study of 376 women with cervical intraepithelial neoplasia. Other studies reported either a weak [61] or null association [58,59].

This inconsistency extended to clinical trials. For example, in a randomized clinical trial, folate supplements (600 μg/day) for two years significantly increased tissue folate and global DNA methylation in twenty post-polypectomy Patients [62]. Similar results were obtained in other clinical trials where folate was administered by patients with colorectal adenoma or carcinoma for shorter periods (3–6 months) [61,63,64,135]. On the other hand, a randomized clinical trial by Cole et al. [102] demonstrated that daily folic acid supplementation (1mg/day) for three to five years did not reduce the risk of adenoma recurrence in 1021 men and women diagnosed with colorectal adenoma. Furthermore, a combined analysis of participants from two randomized clinical trials (Norwegian Vitamin Trial and Western Norway B Vitamin Intervention Trial) demonstrated significant increases in lung cancer risk in participants who administered folic acid (800 μg/day) and vitamin B12 (400 μg/day) [103]. It is worth mentioning that folate doses in these two studies were twice the recommended daily folate intake (400 μg/day) provided in the Dietary Reference Intakes (DRIs) [136] suggesting a biphasic dose-dependent response to folate intake. This assumption is supported by a dose-response meta-analysis by Zhang et al. [137] where a J-shaped correlation between folate intake and breast cancer risk was found. In this meta-analysis, women who consumed 200–320 μg/day were at a lower risk of developing breast cancer; however, women who consumed more than 400 μg/day had a significantly higher breast cancer risk.

Thus far, studies that measured the association between folate intake and global DNA methylation in cancer have yielded inconsistent results as shown above. This inconsistency could be related to the use of varying doses over varying periods; however, a critical confounding factor that should be considered is the type of methylation assays that were used and what they actually measure [138]. Some of these assays measure DNA methylation status in repetitive elements such as LINE (Long Interspersed Nucleotide Element 1), SINE (Short interspersed nuclear elements), and Alu ( Arthrobacter luteus restriction endonuclease) repeats and others examine differentially methylated regions via methylation-sensitive restriction enzymes [139]. Furthermore, some of the assays cover the whole genome while others cover only a certain percentage of the genome [138]. These assays, in general, have different sensitivity and specificity and are prone to over- or under-estimation errors. Details about global DNA methylation analysis methods are beyond the scope of the current review; the reader is directed to a recent article by Kurdyukov et al. [140] that summarizes and compare these methods in terms of outcomes and appropriateness for specific research questions. However, it is worth mentioning that the correlation among these assays is poor, which could mainly be attributed to the fact that they measure different subsets of DNA sequences in diverse regions of the genome [141]. Distinct methylation profiles of various repetitive DNA elements have been found among different tissues and pathological conditions. Furthermore, loss of DNA integrity in cancer may compromise the accuracy of global DNA methylation assay outcome [142]. Thus, using different global DNA methylation assays to evaluate the effect of folate status on DNA methylation could be the reason behind the reported inconsistent and irreproducible findings.

Compared with global DNA methylation, gene-specific methylation analyses in response to folate status have demonstrated more consistent findings. A study by Wallace et al. [56] assessed DNA methylation at specific CPG sites in estrogen receptor alpha (ER-α) and secreted frizzled-related protein-1 (SFRP1) in patients with previous colorectal adenomas who administered folate supplements for three years. In this study, higher levels of folate were associated with higher methylation in the CpG-rich promoters of ER-α and SFRP1; the expression of which has been correlated with an increased risk of colorectal cancer [143,144]. Similarly, promoter methylation of RASSF1A, a proto-oncogene, correlated positively with serum folate concentration in nested cases with malignancies and controls from a prospective study with reported dietary habits and lifestyles [67].

Together, these data suggest that folate might be protective against some cancers via inducing promoter methylation of proto-oncogenes; however, different genes and CpG sites are not equally susceptible to DNA methylation changes in response to folate intake. Accordingly, studies that utilize a candidate gene-specific analysis to investigate the effects of folate on DNA methylation are expected to produce more accurate results than global methylation studies. Furthermore, studies that examine thousands of CpG loci simultaneously will help elucidate the interaction between DNA methylation and folate intake. These studies should take into consideration modifying factors such as age, gender, genetic background, time of exposure (pre-cancer versus post-cancer development), mode of exposure (dietary versus supplemental), and other dietary habits and lifestyles.

### 5.2. Riboflavin, Pyridoxine, and Cobalamin

B vitamins are water-soluble vitamins That are found in a variety of foods such as meat, wholegrains, eggs, dairy products, legumes, nuts, dark leafy vegetables, and fruits (citrus fruits, avocados, and bananas) [136]. There are several types of vitamin B including thiamin (vitamin B1), riboflavin (vitamin B2), niacin (vitamin B3), pantothenic acid, vitamin B6, biotin (vitamin B7), and vitamin B12. While all types of vitamins B are essential in regulating metabolism, hemoglobin synthesis, and maintaining skin and nervous system integrity, vitamins B2, B3, B6 and B12 are specifically critical for one carbon metabolism [145]. Accordingly, the intake of these vitamins may induce DNA methylation and gene expression changes and modify the risk of diseases where DNA methylation play an important role such as cancer. In support of this notion, increased intake of vitamins B2, B6, and B12 by humans have been shown to inversely correlate with cancers such as esophageal cancer [146], cervical intraepithelial neoplasia [76], colorectal cancer [104], and prostate cancer [105]. However, the association between vitamin B levels or administration and DNA methylation was only reported in a few studies that measured the joint effect of vitamin B and folic acid status or included vitamin B in a combined model of other vitamins and micronutrients. Animal studies showed that vitamin B12 deprivation induced global hypomethylation even when combined with a folate-rich diet, which emphasizes the crucial role of vitamin B12 in catalyzing one-carbon metabolism and DNA methylation [147]. This and other animal studies referred to the importance of maintaining a balance between folate and Vitamin B12 in order to preserve physiological DNA methylation patterns [148].

In examining the relationship between vitamin B12 levels and homocysteine levels in the blood, several human studies have reported an inverse association [149,150]. Furthermore, Vitamin B6 and B12 intake was effective in reducing circulating levels of homocysteine [151,152]. These findings along with the one-carbon metabolism pathway we discussed above suggest that B vitamins contribute toward DNA hypermethylation. Nonetheless, studies that reported an association between vitamin B intake and the reduced risk of cancer are inconsistent to whether this protective effect is associated with an induced hypo- or hypermethylation either globally or at the candidate gene level. For example, in a study by Colacino et al. [72], a methylation score for several tumor suppressor genes was calculated and correlated with dietary intake of micronutrients in patients with head and neck cancer. In this study, individuals with the highest quartile of vitamin B12 intake showed significantly less tumor suppressor gene methylation compared with those in the lowest quartile. Additionally, two observational studies have shown that levels of vitamin B12 correlated with global DNA hypomethylation in lung and breast cancer tissues [73,153]. Furthermore, findings that provide minimal to no support of the assumed effect of vitamin B on DNA methylation have been reported. For example, in two separate studies, a lack of any significant changes or correlations between LINE-1 repetitive element methylation and vitamin B levels or supplementations in school-age children or elderly was reported [74,75].

### 5.3. Choline and Betaine

Choline is an essential nutrient that acts as an indirect methyl donor and is involved in several physiological processes such as methylation reactions, cellular membrane integrity, neurotransmitter synthesis, and metabolism [154]. The human body is capable of synthesizing choline in a minimal amount that is not enough to meet the body needs [155]. Thus, the primary source of choline is dietary including fish, poultry, eggs, cruciferous vegetables, and dairy products. The daily recommended intake of choline is 425 and 550 mg for women and men, respectively [136]. Betaine can be obtained from dietary sources or formed inside the body through irreversible oxidation of choline [156]. Betaine is a direct methyl donor; it donates a methyl group to homocysteine resulting in its conversion to methionine [157]. The role of betaine in the methylation process becomes more crucial under conditions of folate deficiency such as excessive alcohol consumption that impairs folate metabolism and interferes with folate-dependent methylation [158,159].

Several studies reported inverse correlations between dietary choline or betaine intake or their plasma levels and homocysteine bioavailability in humans. Melse-Boonstra et al. [160], reported that plasma levels of betaine are significant determinants of fasting homocysteine levels in healthy humans. Cross-Sectional analysis in 1477 women by Chiuve et al. [78], reported lower homocysteine levels in the highest quintile of total betaine and choline intake compared to the lowest quintile. These inverse associations were more pronounced in women who had low folate or high alcohol intake. Similarly, in a study by Lee et al. [161], the association between choline and betaine intake and fasting homocysteine concentrations were evaluated in 1325 males and 1407 females who participated in the Framingham Offspring Study. In this study, higher choline and betaine intakes were associated with lower homocysteine, especially in participants with low plasma levels of folate and vitamin B12. This association was no longer detected in the post-fortification period where most of the products in the United States were fortified with folic acid (140 g folic acid/100 g flour per grain).

In addition to these observational studies, supplementation with either betaine or choline has been shown to reduce homocysteine concentrations in clinical trials. For example, a study by Brouwer et al. [162], reported significant reductions in plasma total homocysteine in 15 healthy participants who administered six grams of anhydrous betaine daily for three weeks. Similar findings were observed in obese subjects who administered betaine (6 g/day) for 12 weeks [79] and healthy men who ingested phosphatidylcholine (2.6 g/day) for two weeks [80]. Doses of betaine and choline that were used in these short-term interventions are much higher than the recommended daily intake we referred to above. Accordingly, further trials that utilize comparable doses to regular daily consumptions are recommended.

Since choline and betaine can act as methyl donors and modify the bioavailability of homocysteine, several studies, mostly animal-based, investigated the association between choline and betaine status and global and candidate gene methylation [3]. Analyses of global and gene-specific DNA methylation in rodent offspring exposed to maternal choline-deficient diet demonstrated global hypomethylation and prompter hypomethylation of CDKN3 (cyclin-dependent kinase 3) [163] calbindin1 [164], VEGF-C (vascular endothelial growth factor-C) and ANGPT2 (angiopoietin 2) genes [165]. Interestingly, in a study by Kovacheva et al. [166], global hypomethylation and gene-specific hypermethylation (insulin-like growth factor-2 (IGF-2)), were observed in rodent offspring exposed to maternal choline-deficient diet. In this study, a concomitant hypomethylation was observed in DNMT1 promoter. These data suggest an early effect of choline deficiency where the lack of methyl groups and impaired one-carbon metabolism result in global hypomethylation that encompasses DNMT1 promoter. This is followed by a phase where the augmented DNMT1 expression subsequently increases DNA methylation at specific gene loci such as IGF-2. This assumption necessitates further investigations; however, if proven right, it may explain the encountered discrepancy among studies that investigated the association between methyl-donating nutrients and DNA methylation status. In this case, factors such as the duration of nutrient intake should be taken into consideration.

Secondary to their role in modifying DNA methylation, choline and betaine status is associated with carcinogenesis [167]. Low choline diet increased levels of S-adenosylhomocysteine in rodents’ livers, reduced DNMT activity, and DNA methylation, and increased the incidence of developing hepatocellular carcinoma [168,169]. While some oncogenes such as c-myc were hypomethylated in this low choline-diet rodent model, the promoter of some tumor suppressor genes such as p53, p16INK4a, PtprO, Cdh1, and Cx26 was hypermethylated [170,171,172]. In support of the role of betaine and choline in protecting against hepatocellular carcinoma, Lupu et al., [173] reported that, in a mouse model, the deletion of betaine-homocysteine methyltransferase gene, which is implicated in choline metabolism resulted in the spontaneous elevation of S-adenosylhomocysteine levels and development of preneoplastic foci in the liver.

In human studies, four case-control studies demonstrated an inverse relationship between choline and betaine intake and the risk of developing breast cancer [106], colon cancer [107], nasopharyngeal cancer [108], and liver cancer [109]. Interestingly, in a nested case-control study within the European Prospective Investigation into Cancer and Nutrition (EPIC) cohort, a significant inverse association between plasma levels of betaine and colorectal cancer risk was observed only in participants with low folate concentrations [110] suggesting that choline and betaine may serve as alternative methyl group donors in states of folate deficiency. A meta-analysis of 11 epidemiological studies supported the protective role of dietary betaine and choline against several types of cancer with the most considerable effect reported for breast cancer followed by nasopharyngeal and lung cancers [174]. In this meta-analysis, it was found that an increment in dietary betaine and choline of 100 mg/day reduced cancer incidence by 11%. In summary, cumulative evidence suggests a significant contribution of choline and betaine to methyl metabolism and DNA methylation and accordingly to gene regulation in carcinogenesis. 

### 5.4. Methionine

Methionine is an essential sulfur-containing amino acid that plays a significant role in regulating metabolism and synthesizing other amino acids, such as cysteine and taurine, and proteins, such as carnitine and melatonin [175,176]. Methionine is not synthesized in the body and should be obtained from foods. While nearly all protein-containing foods have some methionine, eggs, fish, soy, dairy products, and some meats contain high amounts of this amino acid [177]. Methionine is an integral part of the one-carbon metabolism since it serves as a precursor for s-adenosylmethionine, the universal methyl donor for DNA methylation [178]. Thus, dietary intake of methionine is expected to correlate with DNA methylation. However, due to the cyclical nature of the one-carbon metabolism process, methionine excess may have a negative impact on DNA methylation via inhibiting homocysteine methylation to maintain a balance between s-adenosylhomocysteine and s-adenosylmethionine [179,180]. Indeed, there is limited evidence suggesting that dietary methionine induces gene-specific DNA hypermethylation [181]. Furthermore, the effect of methionine is tissue-specific, and studies that measured the correlation between methionine intake and plasma s-adenosyl methionine to s-adenosyl homocysteine ratio have yielded inconsistent findings. For example, three animal studies that have examined the effects of dietary methionine intakes on hepatic levels of methionine, s-adenosyl methionine and s-adenosyl homocysteine demonstrated a significant discrepancy. Finkelstein and Martin [179] reported a lack of induction of hepatic methionine and reduction in the s-adenosyl methionine to s-adenosyl homocysteine ratio in response to methionine-rich diet intake for seven days. In their study, Regina et al. [180] demonstrated a 20-fold increase in hepatic and plasma methionine and 80% increase in the s-adenosyl methionine to s-adenosyl homocysteine ratio in rats fed methionine-rich diet for 14 days. In this study, a lack of increase in the s-adenosyl methionine was observed in the kidney, small intestine, and skeletal muscle. Lastly, Rowling et al. [182] observed an eight-fold increase in hepatic s-adenosyl methionine to s-adenosyl homocysteine ratio in rats fed methionine diet for 10 days. The observed inconsistencies among these studies indicate the complex nature of the effect of methionine that might vary among different tissues and might depend on several factors related to age or duration of exposure.

These contradictory effects might explain the lack of robust correlations between methionine intake and DNA hypermethylation. For example, rats fed a methionine-rich diet showed no changes in p53 [183] or cystathionine beta-synthase promoter methylation [184]. Similarly, in human studies, methionine was associated with decreased methylation levels of RASSF1A, a gene that is implicated in several malignancies [67]. Despite this lack of association between dietary methionine and DNA hypermethylation at the gene level, a positive association at the global level has been observed in mouse gut [185]. In general, there is an evident lack in human studies that measured the effect of dietary methionine on DNA methylation and whether high dietary methionine intake induces DNA hyper- or hypomethylation is still to be determined. Moreover, epidemiologic findings as to whether dietary methionine intake protects against cancer in humans are inconsistent [186]. Higher dietary methionine intake was associated with increased prostate cancer risk (OR = 2.1; 95% CI 1.1–3.9) in men with low Gleason score [187] and failed to demonstrate any association with breast cancer risk in the American Cancer Society Cancer Prevention Study II Nutrition Cohort [111]. Additionally, several ongoing studies are exploiting dietary methionine restriction as a potentiator for the effect of cancer chemotherapy regimen in metastatic cancer [188,189,190], melanoma, and glioma [191]. On the other hand, a meta-analysis of eight prospective studies that measured the association between dietary methionine intake and risk of colon cancer in 431,029 participants reported a summary relative risks (RRs) of 0.77 (95% CI = 0.64–0.92) for the highest versus lowest methionine intake [186]. Additionally, in a dose-response meta-analysis, a linear dose-response relationship was found between methionine intake and the risk of breast cancer; the risk was reduced by 4% for every 1 gram per day increment in dietary methionine intake [192]. In summary, the available findings to date support the need for further investigations to elucidate the direction of the association between dietary methionine and cancer risk and highlight the underlying molecular and epigenetic mechanisms for this potential association.

### 5.5. The Impact of Alcohol and Smoking on Nutrient-Mediated DNA Methylation

Chronic alcohol consumption was a strong predictor of the association between methyl donor nutrient deficiency and the disturbed one-carbon metabolism in several human studies [78,133,158,159,161]. Alcohol has been shown to inhibit methionine synthase activity in the liver resulting in a significant reduction in s-adenosyl methionine levels. Alcohol administration in rodents resulted in a significant decrease in liver concentrations of s-adenosyl methionine and DNA methylation [193]. Additionally, cancer related genes such as c-myc showed increased expression that was accompanied by increased risk of alcoholic liver disease and hepatocellular carcinoma in mice [194,195]. Similarly, tobacco smoke interferes with the one-carbon metabolism and subsequently with the availability of methyl groups required for DNA methylation [196,197,198]. Smoking was also a strong predictor for the association between vitamin B6 and B12 and methylation for several multi-disease-related gene promoters in humans [67]. Additionally, previous cross sectional studies demonstrated that hydrocarbons from tobacco smoke are capable of interacting with vitamin B12 and folic acid resulting in their biological inactivity [199].

### 5.6. The Impact of Early Nutrition in Modulating DNA Methylation

There is growing evidence that maternal nutrition during pregnancy and early postnatal period affects epigenetic profiles of their offspring [200]. Several animal studies reported changes in global and gene-specific DNA methylation in the progeny of animals fed low-protein diet [201,202], caloric-restriction diet [203], or high-fat diet [204,205]. These studies suggest that prenatal or early postnatal periods are critical for the establishment of the epigenome and vulnerable to environmental factors such as nutrition. In support of this assumption, choline supplementation in a maternal murine model modified methionine metabolism genes and global DNA methylation in the offspring [206].

Human studies also supported this assumption and a role of maternal diet in modifying fetal DNA methylation of growth and metabolic genes has been shown in individuals exposed prenatally to famine, and conceivably extreme folate deficiency, during the Dutch Hunger Winter at the end of World War II [207,208]. This association was also supported by Steegers-Theunissen et al. [209], where maternal use of folic acid periconceptually resulted in 4.5% increase in IGF2 methylation and reduced birth weight in children compared to those born to women who had not taken folic acid. Additionally, a study by Ba et al. [69], showed that IGF2 methylation in fetal blood was positively associated with vitamin B12 levels in maternal blood.

Positive associations were observed in the Maternal Nutrition and Offspring’s Epigenome (MANOE) study for maternal intake of betaine and folate and gene-specific DNA methylation in infants, mainly RXRA (retinoid X receptor alpha) gene [210]. Furthermore, the MANOE study demonstrated a time trend for the relationship between methyl donor intake and DNA methylation in pregnant women where women with higher methyl-group intake exhibited higher methylation in the third trimester, and not in the first two semesters [77]. This time-sensitive relationship between methyl donors and DNA methylation in pregnant women might be reflected on their infants’ epigenetic patterns. However, further studies are warranted to investigate this assumption.

In summary, these studies showed the possibility that methyl donor nutrients in the maternal diet could induce DNA methylation levels in the offspring. However, null, even inverse, associations between maternal methyl donor nutrient intake and fetal global or gene-specific DNA methylation have been reported in other clinical studies [70,211,212]. Therefore, recent studies have reported concerns regarding maternal supplementations of folic acid basing their argument on the demonstrated unexpected alterations in normal fetal DNA methylation that could induce detrimental effects [213,214,215,216]. Indeed, the relationship between methyl donors and DNA methylation is complex and may involve other factors such as other dietary components or health status. Accordingly, definitive linear associations of maternal methyl donor intake with fetal DNA methylation could not be confirmed. Furthermore, dietary versus supplemental intake of methyl donors could be a source of inconsistent outcomes. For example, while folic acid deficiency has been clearly shown to be associated with adverse health outcomes, high folate concentrations due to supplementation correlated to mixed outcomes as we summarized above. Accordingly, the currently available evidence is insufficient to determine whether methyl donor supplementation during pregnancy or early postnatal would induce favorable or unfavorable epigenetic effects on the offspring.

## 6. Perspectives of Nutritional Modification of DNA Methylation

Nutrients can regulate several biological processes in our body via modifying epigenetic mechanisms that are critical for gene expression such as DNA methylation. These epigenetic modifications may contribute to the status of our health or the health of our offspring. This dynamic interaction between nutrients we consume throughout our lifetime and our epigenetic signature has now been identified as Nutri-epigenomics. In the current review, we summarized the current literature that reported findings and concepts related to the influence of direct and indirect methyl donor nutrients on DNA methylation and cancer risk. Despite the promising insight Nutri-epigenomics could provide for how to target health from a nutritional standpoint, our knowledge in this topic is still limited and mostly animal-based. Data related to human consumption of methyl donor nutrients were mostly collected from observational studies that are inherently prone to inaccuracy and recall bias. DNA methylation status was assessed by either global methylation approach that lacks a robust clinical relevance or candidate gene-driven approach with limited genome coverage and sensitivity. Further studies in human subjects utilizing sensitive, high-throughput quantitative technologies with a broader range of coverage to systemically analyze the role of methyl donors in modifying DNA methylation and subsequently cancer risk or progression are required. Future work is needed to understand better the interactions among methyl donors involved in one-carbon metabolism and strengthen our understanding of their biological role in health and disease states. The fact that epigenetic marks are potentially reversible and sensitive for nutritional supplementation or pharmaceutical therapies makes the subject of Nutri-epigenomics very attractive, promising, and worthy of study. Yet, we must acknowledge the complexity of the interaction between nutrients and DNA methylation, which is not indicative of a single nutrient. Instead, DNA methylation is a highly regulated process that is gene-sensitive and tissue-dependent and reflects the outcome of several pathobiological processes and environmental exposures. Accordingly, future research should be designed in a way that dissects the effect of single versus combined intake of methyl donors, dietary versus supplementary mode of intake, and dose response relationship. Furthermore, researchers should consider the differential response to methyl donors between healthy tissues and cancerous lesions where aberrant methylation profiles might exist and modify the outcome. Finally, large scale clinical trials in both healthy people and cancer patients are needed in order to provide specific recommendations for methyl donor intake that maintains normal methylation profiles in each group.

## Figures and Tables

**Table 1:** Clinical studies of the impact of methyl donor micronutrients on DNA methylation.

Authors	Population/Tissue	Study Design	Methylation Assay	Conclusion/Outcome
**Folate**				
Wallace et al. [56]	Adults with history of colorectal adenoma Colorectal tissues	Randomized, double-blind controlled trial 1 mg/day for 3 years	Gene-specific quantitative bisulfite pyrosequencing ERα and SFRP1 genes	Higher folate levels were associated with higher levels of ERα (estrogen receptor alpha) and SFRP1 (Secreted Frizzled Related Protein 1) methylation
Pufulete et al. [57]	Colorectal adenoma and cancer patients and heathy controls Colonic tissues	Case-control study Estimates of dietary intake and serum and erythrocyte folate	Global DNA methylation via [(3)H] methyl incorporation	High folate status was associated with decreased plasma homocysteine and increased colonic DNA methylation. Low folate intake and colonic DNA hypomethylation were associated with increased risk for adenoma and cancer
Piyathilake et al. [58]	Patients with cervical intraepithelial neoplasia Cervical tissues	Cross-sectional study Dietary intake pre and post folic acid fortification	Global DNA methylation via Immunohistochemical staining for 5-methyl cytosine	Folic acid fortification did not change global DNA methylation in cells involved in cervical carcinogenesis
Moore et al. [59]	Patients with bladder cancer and controls Blood	Case-control study Dietary intake via food frequency questionnaire (FFQ)	Global DNA methylation via 5-methyl cytosine antibody	Global DNA methylation was significantly lower in cases than control. No significant differences in folate intake between cases and control
Piyathilake et al. [60]	Patients with cervical intraepithelial neoplasia and controls Blood and exfoliated cervical cells	Case-control study Serum levels measured	Global DNA methylation via bisulfite pyrosequencing LINE-1 (Long Interspersed Nucleotide Element 1) analysis	Blood cell (but not cervical cell) DNA was hypomethylated in cases compared to controls and hypermethylated in the highest folate compared to the lowest folate tertile.
Pufulete et al. [61]	Healthy adults Colonic tissues	Cross-sectional study Serum and erythrocyte levels measured	Global DNA methylation via [(3)H] methyl incorporation	Observed weak inverted associations between serum and erythrocyte folate and colonic DNA hypomethylation
O’Reilly et al. [62]	Patients with colorectal adenoma Colonic tissues	Randomized, double-blind controlled trial 600 μg folic acid/day for 6 months	Global DNA methylation via methylation-sensitive restriction enzymes	Folate treatment significantly reversed global DNA hypomethylation in colonic tissues
Cravo et al. [63]	Patients with colorectal adenoma Colonic tissues	Randomized, controlled, cross-over study 5 mg/day for 3 months then switched to placebo for additional 3 months	Global DNA methylation via [(3)H] methyl incorporation	Folate supplementation reversed DNA Hypomethylation, which returned to baseline values after switching to placebo treatment
Kim et al. [64]	Patients with colorectal adenoma Colonic tissues	Randomized, double-blind controlled trial 5 mg/day for 1 year	Global DNA methylation	Folate supplementation increased genomic DNA methylation at 6 months and 1 year
Coppedè et al. [65]	Patients with colorectal cancer Colonic tissues (cancer and adjacent healthy)	Cross-sectional analysis Serum levels were measured	Gene-specific quantitative bisulfite pyrosequencing APC (adenomatous polyposis coli), MGMT (Methylguanine-DNA Methyltransferase), hMLH1 (MutL homolog 1), RASSF1A and CDKN2A (cyclin-dependent kinase 2A) genes	Low folate levels were associated with hMLH1 hypermethylation
Christensen et al. [66]	Breast cancer patients Breast cancer tissues	The Pathways Study: a prospective cohort study Estimates of dietary intake	Genome-wide methylation analysis via Illumina GoldenGate methylation bead-array platform	Higher folate intake was associated with a trend toward increased CpG methylation in several genes
Vineis et al. [67]	Patients with lung cancer and healthy controls Blood	Nested case-control study in The European Prospective Investigation into Cancer and Nutrition (EPIC) Serum levels were measured	Genome-wide quantitative bisulfite pyrosequencing	Folate was associated with increased methylation levels of RASSF1A (Ras association domain family member 1) and MTHFR (methylenetetrahydrofolate reductase)
van Engeland et al. [68]	Patients with colorectal cancer Colorectal biopsies	Netherland Cohort Study (NLCS) Estimated dietary intake via FFQ	Methylation-specific PCR (polymerase chain reaction) for APC-1A (adenomatous polyposis coli-1A), p14(ARF) (alternate reading frame protein of cyclin-dependent kinase 2A), p16(INK4A) (cyclin-dependent kinase inhibitor 4A), hMLH1, O(6)-MGMT (O-6-methylguanine-DNA methyltransferase), and RASSF1A genes	Gene promoters were hypermethylated in patients with low folate intake compared with high folate intake; differences were not statistically significant
Ba et al. [69]	Pregnant women Maternal and cord blood	Cross-sectional study Serum levels were measured	Methylation-specific PCR for IGF2 gene	IGF2 promoter methylation was not associated with serum folate levels in either cord or maternal blood
Hoyo et al. [70]	Pregnant women Cord blood	Cross-sectional study Estimated dietary intake via FFQ	Gene-specific (IGF-2) quantitative bisulfite pyrosequencing	IGF-2 methylation decreased with increasing folate intake
Shelnutt et al. [71]	Healthy non-pregnant women Blood	Folate depletion-repletion clinical trial 115 μg/day for 7 weeks followed by 400 μg/day for additional 7 weeks	Global DNA methylation via [(3)H] methyl incorporation	Observed global DNA hypomethylation during depletion and increases in DNA methylation during repletion
**Vitamin B**				
Colacino et al. [72]	Patients with head and neck cancer	Cross-sectional study Estimated dietary intake via FFQ	Gene-specific methylation analysis via Illumina Goldengate Methylation Cancer Panel	Patients with the highest quartile of vitamin B12 intake showed significantly less tumor suppressor gene methylation compared with those in the lowest quartile
Piyathilake et al. [73]	Patients with lung cancer Cancer tissue and adjacent normal bronchial tissue	Cross-sectional study Tissue levels were measured	Global DNA methylation via [(3)H] methyl incorporation	A direct association was reported between vitamin B-12 and global DNA methylation in cancer tissues but not in normal tissues
Perng et al. [74]	School-age children Blood	Cross-sectional study Plasma levels were measured	Global DNA methylation via bisulfite pyrosequencing LINE-1 analysis	No association between vitamin B12 and global DNA methylation
Hubner, et al. [75]	Old adults Blood	Clinical trial 500 µg folic acid, 500 µg vitamin B12 and 50 mg vitamin B6 for 1 year	Global DNA methylation via bisulfite pyrosequencing LINE-1 analysis	Vitamin B supplementation had no effect on global DNA methylation in blood cells
Piyathilake et al. [76]	Women positive for human papilloma virus Exfoliated cervical cells	Cross-sectional study Plasma levels were measured	Gene-specific (HPV(human papilloma virus)-16) quantitative bisulfite pyrosequencing	Folate and vitamin B12, maintain a high degree of methylation at specific CpG sites in the HPV E6 gene and subsequently reduce the risk of cervical intraepithelial neoplasia
**Choline and betaine**				
Pauwels et al. [77]	Pregnant women Blood	MANOE (MAternal Nutrition and Offspring’s Epigenome) cohort study Estimated dietary intake via FFQ	Global DNA (hydroxy)methylation was measured in blood using LC-MS/MS (liquid chromatography-mass spectrometry/mass spectrometry)	Choline and betaine intake in the first weeks was negatively associated with DNA hydroxymethylation (a step that precedes demethylation)
Chiuve et al. [78]	Healthy women Blood	Cross-sectional study from The Nurses’ Health Study (NHS) Estimated dietary intake via FFQ	Plasma total homocysteine measurement via HPLC (high performance liquid chromatography)	Total choline + betaine intake was inversely associated with homocysteine (measured as a surrogate biomarker for effective methyl donation and DNMT activity)
Schwab et al. [79]	Obese adults Blood	Randomized, double-blind controlled trial Betaine supplements (6 gm/day) for 12 weeks	Plasma total homocysteine measurement via HPLC	Betaine supplementation decreased the plasma homocysteine concentration
Olthof et al. [80]	Healthy men Blood	Randomized, double-blind controlled trial Choline supplements (2.6 gm/day) for 2 weeks	Plasma total homocysteine measurement via HPLC	Choline supplementation decreased the plasma homocysteine concentration
**Methionine**				
Vineis et al. [67]	Details are in the folate section of the table			Methionine was associated with decreased methylation of RASSF1A gene
Pauwels et al. [77]	Details are in the choline and betaine section of the table			A high intake of methionine showed lower DNA hydroxymethylation (a step that precedes demethylation)
Perng et al. [81]	Healthy adults Blood	Multi-Ethnic Study of Atherosclerosis (MESA) Stress Study Estimated dietary intake via FFQ	Global DNA methylation via bisulfite pyrosequencing LINE-1 analysis	Dietary methionine was not associated with global DNA methylation
Tao et al. [82]	Breast cancer patients and control Breast cancer tissue	Cross-sectional study from the Western New York Exposures and Breast Cancer Study (WEB Study) Estimated dietary intake via FFQ	Methylation-specific PCR of E-cadherin, p16, and RAR-β(2) (retinoic acid receptor beta 2) genes	Dietary intake of methionine was not associated with promoter methylation of E-cadherin, p16, and RAR-β(2) genes

**Table 2:** Clinical studies of the impact of methyl donor micronutrients on cancer.

Authors	Population/Tissue	Study Design	Conclusion/Outcome
**Folate**			
Giovannucci et al. [83]	Male and female adults	The Nurses’ Health Study, and the Health Professionals Follow-up Study Estimated dietary intake via FFQ	High dietary folate was inversely associated with risk of colorectal adenoma in women and men
Su et al. [84]	Male and female adults	The NHANES I Epidemiologic Follow-up Study (NHEFS) Estimated dietary intake via FFQ	Significant association between folate intake and lower risk of colon cancer among men and non-alcohol drinkers, but not women or alcohol drinkers
Fuchs et al. [85]	Female adult	The Nurses’ Health Study Estimated dietary intake via FFQ	Higher folate intake reduces the risk of colon cancer associated with a family history of the disease.
Stevens et al. [86]	Male adult	The American Cancer Society Cancer Prevention Study II Nutrition Cohort Estimated dietary intake via FFQ	Higher intake of folate was associated with a nonsignificant decrease in the risk of advanced prostate cancer
Gylling et al. [87]	Patients with colorectal cancer and matched controls	The Nurses’ Health Study	Low plasma levels of folate were associated with a reduced risk of colorectal cancer
Giovannucci et al. [88]	Female adult	A nested case-control study in the population-based Northern Sweden Health and Disease Study Estimated dietary intake via FFQ	Folate intake was associated with a lower risk for colon cancer
Konings et al. [89]	Male and female adults	The Netherlands Cohort Study Estimated dietary intake via FFQ	The study reported an inverse association between colon cancer risk and total dietary folate intake.
Terry et al. [90]	Patients with colorectal cancer and matched controls	A nested case-control study in the Canadian National Breast Screening Study Estimated dietary intake via FFQ	Folate intake was inversely associated with the risk of colorectal cancer
Wei et al. [91]	Patients with colorectal cancer and matched controls	A nested case-control study in the Nurses’ Health Study (NHS) and the Health Professionals Follow Up Study (HPFS) Estimated dietary intake via FFQ	Folate intake was associated with lower risk of colon cancer; however, rectal cancer cases tended to have slightly higher folate
Harnack et al. [92]	Female adults	Population-based Iowa Women’s Health Study cohort Estimated dietary intake via FFQ	There were no independent associations of folate with incidence of colon cancer; however, relative risk was lower among those who had a combined high folate and high vitamin B-12 or high folate and vitamin B6.
Benito et al. [93]	Colorectal cancer and matched controls	A case-control study Estimated dietary intake via FFQ	Folate intake was associated with reduced risk of colorectal cancer
Ferraroni et al. [94]	Colorectal cancer and matched controls	A case-control study Estimated dietary intake via FFQ	There was a trend of a protective effect of high folate intake against colorectal cancer development
Freudenheim et al. [95]	Colorectal cancer and matched controls	A case-control study Estimated dietary intake via FFQ	Folate intake was associated with a reduced risk of rectal cancer but not colon cancer
Glynn et al. [96]	Patients with colorectal cancer and matched control	A nested case-control study within the Alpha-Tocopherol Beta-Carotene Study cohort of male smokers Estimated dietary intake via FFQ and serum levels were measured	No association between serum folate and colorectal cancer. High dietary folate intake was protective against colorectal cancer.
La Vecchia et al. [97]	Patients with colorectal cancer and matched control	Case-control study Estimated dietary intake via FFQ	No association between dietary folate and risk of colorectal cancer
Le Marchand et al. [98]	Patients with colorectal cancer and matched control	Case-control study Estimated dietary intake via FFQ	Decreased risk of colorectal cancer in subjects who consume high levels of folate and vitamin B6
Levi et al. [99]	Patients with colorectal cancer and matched control	Case-control study Estimated dietary intake via FFQ	No significant association between folate intake and colorectal cancer
Boutron-Ruault et al. [100]	Patients with colorectal cancer and matched control	Case-control study Estimated dietary intake via FFQ	Folate intake prevents adenoma formation and protective against adenoma growth associated with alcohol
Kato et al. [101]	Patients with colorectal cancer and matched control	A nested case-control study in the New York University Women’s Health Study cohort Serum levels were measured	The risk of colorectal cancer in the subjects in the highest quartile of serum folate concentrations was half that of those in the lowest quartile
Cole et al. [102]	Patients with colorectal adenoma	Randomized, double-blind controlled trial 1 mg/day of folic acid for 3 years	Folic acid at 1 mg/day does not reduce the risk of colorectal adenomas or their advancement to neoplastic lesions
Ebbing et al. [103]	Male and female adults with ischemic heart disease	Norwegian Vitamin Trial and Western Norway B Vitamin Intervention Trial folic acid (0.8 mg/day) plus vitamin B12 (0.4 mg/day) for 6–7 years	Folic acid plus vitamin B12 supplementations were associated with increased cancer outcomes and all-cause mortality in patients with ischemic heart disease
**Vitamin B**			
Otani et al. [104]	Patients with colorectal cancer and matched control	Case-control study Estimated dietary intake via FFQ	Neither vitamin B2, vitamin B6, nor vitamin B12 were significantly associated with colorectal cancer
Hultdin et al. [105]	Patients with prostate cancer and matched control	Case-control study Serum levels were measured	Serum concentrations of vitamin B12 were associated with an up to three-fold increase in prostate cancer risk
Gylling et al. [87]	Details are in the folate section of the table		Plasma levels of vitamin B12 were inversely associated with rectal cancer risk
**Choline and Betaine**			
Du et al. [106]	Patients with breast cancer and matched control	A hospital-based case-control study Serum levels were measured	Serum betaine but not choline was inversely associated with risk of breast cancer development in subjects with below-median dietary folate intake
Lu et al. [107]	Patients with colorectal cancer and matched control	Case-control study Estimated dietary intake via FFQ	Total choline intake was inversely associated with colorectal cancer risk however no significant associations were observed for betaine or total choline plus betaine intakes
Zeng et al. [108]	Patients with nasopharyngeal cancer and matched control	Case-control study Estimated dietary intake via FFQ	Intakes of total choline, betaine, and combined choline and betaine were inversely associated with nasopharyngeal cancer
Zhou et al. [109]	Patients with liver cancer and matched control	Case-control study Estimated dietary intake via FFQ	Higher intake of choline and betaine was associated with a lower risk of liver cancer
Nitter et al. [110]	Patients with colorectal cancer and matched control	A nested case-control study within the European Prospective Investigation into Cancer and Nutrition (EPIC) Plasma concentrations were measured	Higher betaine and choline concentrations were associated with lower risk of colorectal cancer especially in subjects with lower folate concentrations
**Methionine**			
Feigelson et al. [111]	Patients with prostate cancer and matched control	Case-control study Estimated dietary intake via FFQ	A direct association between higher methionine intake and prostate cancer risk was observed only in men who have at least one MTHFR A1298C allele
Giovannucci et al. [83]	Details are in the folate section of the table		Methionine intake was inversely associated with risk of having larger adenomas (1 cm or larger)
Su et al. [84]	Details are in the folate section of the table		Significantly increased risk of colon cancer in men who consume low-methionine diet compared to those who consume high methionine diet
Fuchs et al. [85]	Details are in the folate section of the table		Higher intake of methionine reduces the risk of colon cancer associated with a family history of the disease
Nitter et al. [110]	Details are in the betaine and choline section of the table		Methionine concentrations were inversely associated with colorectal cancer risk with borderline significance
